# Engaging with Socio-Economically Disadvantaged Communities and Their Cats: Human Behaviour Change for Animal and Human Benefit

**DOI:** 10.3390/ani9040175

**Published:** 2019-04-17

**Authors:** Jenni L. McDonald, Jane Clements

**Affiliations:** 1 Veterinary Department, Cats Protection, National Cat Centre, Haywards Heath RH17 7TT, UK; jane.clements@cats.org.uk; 2 Bristol Veterinary School, University of Bristol, Bristol BS40 5DU, UK

**Keywords:** unowned cats, Trap-Neuter-Return (TNR), behaviour change, *Felis catus*, community engagement, stray cat, deprived areas

## Abstract

**Simple Summary:**

The overpopulation of domestic cats (*Felis catus*) is an important welfare concern and can be a particular problem in socio-economically deprived areas. Trap-Neuter-Return (TNR) activities are a humane way of managing unowned cat populations; however continued movement of cats into an area can derail the TNR effort. Consequently, for sustainable change, it is recommended that TNR activities are linked with community engagement to encourage positive behaviours towards cats, such as continued reporting of unowned cats for neutering. We investigated the impact of a community-based partnership approach to TNR, in order to (1) determine the acceptability of the project within the community; (2) determine whether the project leads to sustainable behaviour change and (3) assess the potential benefits of participation in activities. We found residents had increased self-efficacy and confidence to help the cats within the community and were more likely to report unowned cats for neutering now compared to previous years. Engaging communities with programs to control cat numbers, can effectively overcome barriers to helping cats. We hope that the promising results from this program will encourage future efforts to consider community participation when cat management is being delivered, to overcome barriers to helping cats in often hard-to-reach populations.

**Abstract:**

The overpopulation of free-roaming domestic cats (*Felis catus*) is fuelled by uncontrolled breeding of both owned and unowned populations and has been identified as a particular problem in socio-economically deprived areas. Consequently, for sustainable change, it is recommended that Trap-Neuter-Return activities are linked with community engagement to encourage positive behaviours towards cats. This paper assesses the acceptability and impact of a community-partnership program called “Bulwell Cat Watch” (BCW), set-up to control cat numbers in Bulwell, UK. The data are based on a (1) cross-sectional survey (*n* = 478); (2) pre-post analysis (*n* = 21); and (3) targeted survey of people known to engage with BCW (*n* = 34). We found significant associations between awareness of BCW and an increased likelihood of reporting unowned cats now compared to previous years. Respondents reported increased self-efficacy and confidence to help cats. Our pre-post study corroborated these findings with residents significantly more likely to report unowned cats compared to when surveyed pre-BCW. An indirect benefit to residents engaged with the program was the positive impact on confidence and self-esteem. Taken in combination these results show community partnerships can effectively engage often hard-to-reach populations and foster sustainable management by overcoming barriers to helping cats, alongside the potential for wider community benefits.

## 1. Introduction

The ability of domestic cats (*Felis catus*) to revert to a free-living status paired with their nature as prolific breeders has led to large numbers of unowned cats across the world, including in the UK. These cats experience a vulnerable life outside, and are often regarded as a nuisance and threat to public health [1,2,3]. Limiting their ability to reproduce with trap-neuter-return (TNR) activities is recognised as the most humane way of managing unowned cat populations [4] and improving their welfare [5,6]. However, the use of TNR is often challenged due to conflicting reports of its efficiency at reducing unowned cat populations [7,8,9]. Continued immigration of cats and abandonment of owned pets can render TNR efforts less effective [10]. Consequently, for sustainable change, it is recommended that TNR activities be linked with community engagement to encourage uptake of positive behaviours towards cats, such as continued reporting of unowned cats for neutering and controlled breeding of the owned population to prevent unwanted litters [11]. However, to date most TNR strategies in the UK are largely reactive with limited application and evaluation of a community-partnership approach.

The overpopulation of unowned cats, although difficult to quantify, is a problem in many parts of the world including the UK [1] where large numbers of cats enter rescue organisations annually [12,13]. Previous work suggests that overpopulation is a particular problem in urban areas of socioeconomic deprivation [14,15] with socioeconomic factors found to influence human behaviours that may contribute to the problem [16,17]. In these circumstances, there can be many barriers to engaging in positive behaviour, even when individuals may be motivated to do so. For example, restricted funds will affect people’s ability to afford to neuter cats resulting in lower neutering and higher pregnancy rates [17]. The reporting of stray cats for neutering is highly desirable, yet it is likely that there is an under-representation of people from socio-economically deprived communities engaging in these activities, with low uptake in comparable wildlife recording schemes [18]. Where a community suffers from multiple disadvantages, the effects manifest in poor outcomes for human wellbeing, increased barriers to positive behaviour change and will in turn be demonstrated through lower levels of animal welfare [19].

Effective routes of engagement within socio-economically deprived communities will be paramount to tackling feline overpopulation, with blanket messages and mass marketing likely to be ineffective [11,20]. Previous work tackling health-related problems in disadvantaged communities have found a range of barriers to individuals’ motivation to participate, including, but not limited to; a lack of social support networks, low feelings of personal agency and autonomy and distrust of service providers [21]. Consequently, communities can be disengaged [22] and ‘hard to reach’, resulting in low uptake of particular schemes [18]. Previous work has highlighted how behaviour change science, tailoring communications and creating the right opportunities, can be used to help overcome barriers [23,24] and curb the feline overpopulation problem [11].

Here, we explore the theory that a community-based partnership approach to TNR leads to sustainable behaviour change within a socio-economically deprived community. The objectives of our research were threefold: (1) to determine the acceptability of the project within the community; (2) to determine whether the project leads to sustainable behaviour change and (3) to assess the potential benefits of participation in activities. We addressed these objectives from the combined results of multiple evaluation surveys. First, we used a cross-sectional questionnaire to assess the extent to which behaviours have changed within the community and potential links to the project. Second, we used pre-post surveys to evaluate whether the same individuals have changed their behaviour since the project. Third, we considered the impact of the project from the perspective of residents who have proactively engaged.

## 2. Materials and Methods

### 2.1. The Community Engagement Program

Bulwell, Nottingham is the site of “Bulwell Cat Watch” (BCW), a community-partnership project set-up by Cats Protection, a UK feline welfare charity, to control cat numbers. Bulwell falls within the 10% most deprived areas in the UK [25], and consists of approximately 8000 households.

The project launched in September 2016 with a door to door survey of 10% of households (*n* = 776) to assess views on unowned cats, neutering and to identify locations of unowned cats (for more details see [11]). Other methods to engage with and involve the community were also employed; focus groups with stakeholders and residents, interviews with key workers and meetings with police and housing associations. These interventions involved the community in the project from the outset and raised awareness of TNR activities.

The BCW project used the COM-B system which provides a simple model that can be applied to any behaviour in any context to explain the basic interacting elements involved in behaviour change [26]. The Behaviour Change Wheel model (of which the COM-B systems sits at the core) combines elements from previous frameworks of behaviour change and helped inform BCW interventions (see [11] for further details). Additionally, survey responses improved understanding of the residents in terms of their attitudes and knowledge about unowned cats and finding out what the barriers might be in helping them. This allowed the project messaging to speak to the community using language that identified with them, according to their concerns and values. For a detailed description of the framework see [11].

Multiple routes of engagement were put in place to encourage residents to report stray cats. Means of communicating with the outreach team, and for the community to interact with each other, were facilitated through community hub drop in sessions, a digital application, a Facebook group, a range of events, door knocking, leafletting, and posters.

Engagement ran alongside TNR operations, with community reporting of unowned cats providing the intelligence for targeted TNR to commence in November 2016. Between November 2016 and July 2018 BCW received 360 reports of unowned cats from the community. Within the same time-frame, the intelligence from reports resulted in 105 unowned cats trapped as part of TNR operations, an additional 59 unowned cats rehomed and seven lost cats reunited with their owners.

### 2.2. Evaluation Surveys and Analysis

A series of surveys were developed to assess the impact of BCW on human behaviour within the community, specifically the reporting of unowned cats for neutering. These were run in August 2018, two years after BCW first launched. Evaluation of BCW was approved by University of Bristol Faculty of Health Science Research Ethics Committee approval number 38661.

#### 2.2.1. Cross-Sectional Survey

The questionnaire, called the “Bulwell community questionnaire” (BCQ), was developed to evaluate whether any change in behaviour towards unowned cats in the community was linked to awareness of BCW. The BCQ was distributed online via Facebook advertisements targeted at people in Bulwell and paper copies which were handed out at community events.

The BCQ had a “free answer” section, where respondents were asked whether they knew two actions they could take to help unowned cats. They were subsequently asked if they had carried out either of the actions and when they first carried out this action. This provided an unprompted measure of the awareness respondents had about human behaviour that helps cats and if these were new behaviours that had developed since BCW launched. A content analysis of responses in NVivo Pro 11 [27] enabled behaviours to be categorised into key behavioural groups.

The BCQ obtained further data on behaviours around unowned cats using close-ended questioning, this included whether respondents had reported unowned cats, were likely to report unowned cats and whether this behaviour had changed over the past two years.

Behavioural differences (knowledge, intentions, actions and changes) were compared between respondents that were aware of BCW and those not aware of BCW, using the chi-square test in R [28].

To assess the community’s views on BCW we included a free text box, in addition to specific questions about whether BCW improved their confidence and ability to help unowned cats, to get a measure of empowerment and self-efficacy respectively. A qualitative analysis of responses in NVivo Pro 11 [27] identified key themes from the free-text responses.

#### 2.2.2. Pre-Post Survey

This questionnaire was developed to evaluate direct engagement efforts by re-surveying the same community members that were randomly selected in 2016 prior to commencement of BCW, providing a pre-post measure of the knowledge and behaviour towards unowned cats of the same individuals. Knowledge, behavioural intentions and reported behaviour were compared between pre-BCW surveys and post-BCW surveys using the McNemar chi-square test in R [28]. See [11] for further details of the initial survey.

#### 2.2.3. Targeted Survey

Further data were collected from a targeted questionnaire aimed at residents that were known to actively engage and/or volunteer with BCW. This was posted online on the BCW Facebook group page and paper copies were handed out to residents known to engage with BCW. This questionnaire was developed to ascertain the individual benefits and outcomes of taking a more proactive role in BCW. Data analyses were descriptive.

## 3. Results

### 3.1. A Cross-Sectional Survey of the Community

#### 3.1.1. Behaviours towards Unowned Cats

An online (*n* = 457) and paper (*n* = 21) survey was distributed to residents living in the Bulwell area. 86% (*n* = 409) of respondents cited behaviours they perceived to help unowned cats, totalling 882 behaviours. Thematic analysis revealed the most common behaviours reported were provision of food, adoption, neutering and reporting (see Table 1 for list of commonly cited behaviours).

Survey responses indicated that respondents who were aware of BCW were more likely to cite reporting unowned cats as a way to help them compared to respondents who were unaware of BCW (24% vs. 15% respectively; Χ^2^ = 4.02, df = 1, *p* = 0.04). Additionally, respondents that were aware of BCW were also more likely to have started reporting cats in the past two years compared to respondents who were unaware of BCW (10% vs. 4% respectively; Χ^2^ = 5.69, df = 1, *p* = 0.017).

This result is corroborated from the close-ended survey questions; responses indicated that respondents that were aware of BCW were significantly more likely to have reported unowned cats for neutering, had increased likelihood of doing so in the future and were also more likely to report a positive change in the likelihood of helping cats (Table 2).

78% of people that had heard of BCW said it made them feel more confident in reporting stray cats and 75% reported that their ability to help stray cats had improved due to BCW.

#### 3.1.2. Views on the Engagement Program

Views on the BCW program were coded from 110 responses, comprising 79% of people that were aware of BCW with the remaining respondents not providing an answer to this free text question. The majority (95%) of responses contained positive view points, 7% contained negative comments and 2% contained neutral statements. Note these measures are non-exclusive, whereby some individuals provided both positive and negative comments.

The majority (44%) of positive comments were unspecific, e.g., “good work”, “fantastic people”, “very helpful”; 34% of comments made specific reference to helping and improving the welfare of unowned cats for example:

“they help the strays that no one is interested in…”

15% of comments also referred to helping people and/or the community to help cats for example:

“helps out cats by encouraging the community to work together…”

“They were very helpful and kind when helping us get our stray cat Marty in to be looked at…”

The eight negative comments centred around three main themes. First, the difficulty in ascertaining whether a cat is unowned (*n* = 3).

*“It sounds like a good idea but how can you tell if a cat is unowned or just uncared for?”*

Second, whether the project is having an impact (*n* = 3).

*“A great idea, if it works in practice.”*

Third, a perceived lack of need (*n* = 2).

*“I think it is a good scheme, however I have never encountered a problem with unowned cats but if I was to do so I would help!”*

### 3.2. Pre-Post Survey

A follow-up survey was sent to individual respondents who had completed the survey in 2016 (pre-BCW), who had consented to be contacted and who had provided identifiable contact details, resulting in 136 individuals. Responses were received back from 21 individuals, equating to a response rate of 15%.

Due to a small sample size the power to detect any changes is limited; however, we did find some significant changes. First, significantly more respondents considered everyone in the community with an interest in cats to be responsible for looking after unowned cats (Table 3). Second, significantly more respondents provided food for unowned cats (Table 3). Third, significantly more respondents reported that they would be likely to arrange or take unneutered unowned cats to get neutering (Table 3). This increase is also corroborated by 57% (*n* = 12) of respondents stating they are more likely to report unowned cats now compared to previous years.

### 3.3. Active Engagement

Bulwell Cat Watch created opportunities for the residents to engage with program, both within various registered roles and unregistered volunteers such as cat caretakers and members of the BCW Facebook community.

A survey targeting residents that have actively participated with BCW provided insights from 34 respondents into why they participate and the perceived benefits. Participants engaged with BCW through the following activities (note participants were often engaged in more than one activity):

- 20 provided food for unowned cats;
- 18 posted on BCW Facebook page;
- 17 provided shelter for unowned cats;
- 12 attended a BCW event;
- 11 reported unowned cats;
- 9 volunteered with BCW team.

Volunteering or engaging with the project improved the perceived confidence (91%) and ability (85%) of respondents to help unowned cats.

*“Volunteering with Cats Protection is very rewarding and I hope to be volunteering and helping cats and local communities for many years.”*

*“Without Bulwell cat watch I would not have been able to help as much as I wanted to they provided me with outdoor beds and help.”*

#### Broader Benefits of Participating

91% of respondents reported enjoyment and a sense of personal achievement as outcomes of engaging with the program. Additionally, broadening of life experience, meeting friends and learning new skills were also linked with engagement activities by more than 40% of respondents.

Participation played a role in individual development; 47% said it increased their sense of community, and 26% said it increased their confidence and self-esteem.

*“Bulwell cat protection has given me and my community the opportunity to control the epidemic of stray cats and kittens in my community. Much appreciated…”*

*“It really improved my belief in the care of the cats in the community…”*

## 4. Discussion

Sustainable management of the urban unowned cat population is dependent upon community members practicing responsible behaviours toward unowned and owned cats, yet few TNR operations attempt to bring about behaviour change within communities. In urban socio-economically deprived communities, the barriers to engagement are often greater [18,23], yet it is in these communities where unowned cats are most likely to be [14,15,17]. However, barriers to engagement are not insurmountable. Engagement paired with TNR can be well received by residents and result in reports of unowned cats for neutering. Multiple strands of evidence support that working with communities alongside TNR can result in positive behaviour change towards unowned cats.

Continued reporting and neutering of immigrant cats is key to sustaining the long-term benefits of TNR [29], with even low levels of immigration significantly reducing the effectiveness of any management program [10,30]. We find an association between awareness of BCW and an uptake of reporting behaviour. Although we would expect respondents that help unowned cats to, by their nature, also be more aware of BCW, we contend that it is the change in behaviour that provides evidence that BCW may have a more causative, opposed to correlative, role. This result is further corroborated by the pre-post survey. Despite a small sample size, the ability to evaluate whether behaviour of the same individuals have changed through time provided additional insight into the potential causative impact of BCW. Residents were more likely to report cats two years after BCW launched compared to before BCW. Consequently, the establishment of these reported positive behaviours within the community will hopefully lead to continued reporting and timely neutering of unowned cats by residents.

A sense of community has been identified as an important cause of behaviour change in health-related studies [31,32]. The present study showed that a community-focused approach was viewed positively and almost half of the volunteers experienced an increased sense of community by participating. Increasing the community-feel of the project, both through online and face-to-face engagement, provided novel networks that may have influenced the likelihood of behaviour change through various means including; increasing exposure to, and transmission of, social norms related to behaviours that help cats; improvements to psychological mechanisms such as self-esteem and self-efficacy; and access to resources. Online engagement was one popular route of participation, due to the creation of a new and lasting network through a Facebook page. Social networking is often used as a tool for engagement in health-related programs, with varying levels of success. Creation of an online Facebook community fosters interactivity among users, encouraged user driven content [33] and can lead to further dissemination of information across networks.

It has long been thought that cognitive evaluations are influential on a person’s perceived capabilities [34], with confidence an important barrier to participation in community schemes, especially in socio-economically deprived communities [18]. Importantly, our research indicates that a program of engagement can increase a person’s perceived confidence and ability to report unowned cats. These measures of self-efficacy and confidence may mediate between intentions and behaviour [35,36], where a positive “can do” cognition can be the difference between someone intending to help cats to actually helping unowned cats. Therefore, it is unsurprising that we find an increase in both intentions and behaviour to report unowned cats.

This project provided multiple routes for residents to report unowned cats. Although multi-channel approaches are valuable as they account for diverse preferences of community members, we found positive comments around BCW were centred around the team in the community. This could reflect a preference for more personalised and tailored routes of engagement, perhaps as they are perceived as a more trusted source of information, can be tailored to individuals and are less restricted by financial or social barriers. All of these have been identified as potential barriers to engagement with hard-to-reach groups in other scenarios [18,20,23].

Going forward, more intensive collection of qualitative data is required to explore how specific interventions have helped individuals overcome barriers and whether the efficiency of such interventions is dependent upon the traits and/or demographics of individuals. Research into the mechanisms that overcome barriers to behaviour change across a wider range of communities and demographics, will lend support to our understanding of how engagement can most effectively help communities. Additionally, the ‘success’ of such interventions will not just be from cat reporting rates but from wider long-term personal and community benefits.

The project provided avenues for more engaged members of the community to become involved. Similar to volunteering in other contexts [37], our study indicates significant personal benefits for individuals engaged with BCW, including a sense of community, enjoyment and personal achievement. Such feelings are valuable in communities where human wellbeing is considered to be low. In turn, this positive psychology [38] is promising for positive action towards animal welfare [19]. Indeed, the surveyed participants reported increased capability and confidence to help cats. Empowering individuals to take a more active role both online and within the community secures cat advocates within the community to safeguard the operations of the project in the long-term. This offers a potential new type of volunteer model; whereby empowered community members can be potential guardians for vulnerable cats. The long-term impact of such roles on cat welfare remains to be seen. Additionally, increased confidence and self-esteem were reported by some participants, which has the potential to open up other opportunities to these engaged individuals. Future efforts exploring these wider benefits of community engagement across a range of individuals would be valuable.

Negative perceptions of BCW were limited and variously attributed to difficulties identifying unowned cats, questioning the impact of the project and a perceived lack of need. Although it is unlikely that messaging will reach all within a community, awareness of these concerns can enable effective mitigation against them going forward. First, explanation of the careful procedures used to identify a cat as unowned may help address concerns regarding the potential to misidentify an owned cat as unowned. Second, the measure of impact will be subjective to the individual, for example it may be welfare orientated for some and for others it may be based on observed reductions in unowned cats. Also, the perceived impact of the project is likely to be greatest in areas where unowned cats were more abundant. Consequently, explaining the benefits of the project may increase views on impact in some cases but not all. Qualitative data on perceptions of impact would be an interesting area for future studies. Third, unowned cat densities can vary dramatically even across a short distance [8], therefore some residents may not perceive a problem within their area. In such cases disengagement may be unsurprising due to difficulties in conceptualizing a problem they do not experience themselves. In these situations, a community-focused approach to raise awareness will be of benefit.

This study has several limitations. One drawback is the online survey, as self-selecting the data obtained through internet surveys may be prone to bias [39] and it is not possible to estimate response rates. Consequently, we are unable to infer the true prevalence of behaviours within the community. Despite this limitation, online surveys allow for snowball sampling, a cost-effective way of reaching out to a vast array of respondents. This allows for a large enough sample size to explore trends in behaviour through time, despite not being able to identify baseline rates. A second drawback of this approach is that behaviour change is measured via survey responses opposed to direct monitoring in the field. However, 360 reports of unowned cats were received by the BCW team during the two years prior to the evaluation surveys being run indicating community engagement with reporting behaviour, despite not being able to monitor individual behaviour per se. Additionally, this work cannot infer the sustainability of the behaviour change through time, requiring more longitudinal studies. Despite these limitations, the information collated was sufficient to provide comparable and significant results across the different modes of data collection, which we hope will motivate similar studies across other areas.

## 5. Conclusions

TNR is likely to have limited short-term success if used as a one-off intervention. The actions within communities are integral to the long-term welfare of cats, with continued reporting of unowned cats key to prevent overpopulation of cats returning. Working with communities through a variety of engagement routes can encourage people to report unowned cats. One of the success stories of BCW is increasing the involvement of community members in reporting unowned cats. These interventions proved to be the building blocks of a framework which created social support, increased self-efficacy and feelings of empowerment to help the cats. We hope that the promising results from this program will motivate future efforts to consider community engagement when cat management is being delivered, to overcome barriers to helping cats in often hard-to-reach populations.

## Figures and Tables

**Table 1:** Most commonly cited behaviours perceived to help unowned cats from free-text survey questioning.

Behaviour Cited	Percentage (*n* = 882 Behaviours)
Provide food	20%
Adopt a stray	17%
Neuter	13%
Report them to a charity or organisation perceived to be able to help	13%
Provide water	8%
Provide shelter	8%
Trap them	5%
Provide veterinary care	4%

**Table 2:** Results from close-ended questions on behaviours (actual, intentions and change in intentions) around reporting of unowned cats.

Classification	Question	Aware of BCW (*n* = 139)	Not Aware of BCW Watch (*n* = 330)	Significance
Actual behaviour	Have provided or helped arrange neutering for unowned cats e.g., by reporting cats to charity.	30% (*n* = 42)	13% (*n* = 44)	Χ^2^ = 17.51, df = 1, *p* < 0.001
Behavioural intentions	Likely to take or arrange neutering for unowned cats e.g., by reporting cats to charity.	86% (*n* = 120)	72% (*n* = 237)	Χ^2^ = 10.55, df = 1, *p* = 0.001
Change in behavioural intentions	More likely to report unowned cats now compared to previous years.	68% (*n* = 95)	56% (*n* = 186)	Χ^2^ = 5.39, df = 1, *p* = 0.02

**Table 3:** Differences in reported knowledge, behaviour and behavioural intentions pre-BCW and post-BCW and any associated significance.

Classification	Question	Pre-BCW Survey	Post-BCW Survey	Significance
Knowledge	Disagree that related cats will not mate with each other.	52% (*n* = 11)	57% (*n* = 12)	Χ^2^ = 0, df = 1, *p* = 1
	Agree that neutering reduces anti-social behaviour, such as wailing and spraying.	62% (*n* = 13)	52% (*n* = 11)	Χ^2^ = 0.1, df = 1, *p* = 0.75
	Disagree that female cats should be allowed to have kittens before being neutered.	52% (*n* = 11)	67% (*n* = 14)	Χ^2^ = 0.8, df = 1, *p* = 0.37
	Everyone in the community are responsible for looking after unowned cats.	24% (*n* = 5)	38% (*n* = 8)	Χ^2^ = 0.36, df = 1, *p* = 0.55
	Those in the community with an interest in the cats are responsible for looking after unowned cats.	9% (*n* = 2)	57% (*n* = 12)	Χ^2^ = 6.75, df = 1, *p* = 0.009 *
	It is important that unowned cats are provided with shelter.	67% (*n* = 14)	86% (*n* = 18)	Χ^2^ = 1.5, df = 1, *p* = 0.22
	It is important that unowned cats are neutered.	90% (*n* = 19)	90% (*n* = 19)	Χ^2^ = 0, df = 1, *p* = 1
Behaviour	Have provided food for unowned cats.	24% (*n* = 5)	62% (*n* = 13)	Χ^2^ = 6.12, df = 1, *p* = 0.01 *
	Have provided shelter for unowned cats.	24% (*n* = 5)	29% (*n* = 6)	Χ^2^ = 0, df = 1, *p* = 1
	Have provided water or milk for unowned cats.	24% (*n* = 5)	62% (*n* = 13)	Χ^2^ = 3.5, df = 1, *p* = 0.06
	Have provided vet treatment for unowned cats.	9% (*n* = 2)	9% (*n* = 2)	Χ^2^ = 0, df = 1, *p* = 1
	Have provided vaccinations for unowned cats.	9% (*n* = 2)	9% (*n* = 2)	Χ^2^ = 0, df = 1, *p* = 1
	Have provided or helped arrange neutering for unowned cats e.g., by reporting cats to charity.	9% (*n* = 2)	29% (*n* = 6)	Χ^2^ = 1.1, df = 1, *p* = 0.29
Behavioural intentions	Likely to take or arrange neutering for unowned cats e.g., by reporting cats to charity.	29% (*n* = 6)	76% (*n* = 16)	Χ^2^ = 8.1, df = 1, *p* = 0.004 *

* Significant (*P* < 0.05) results.

## References

[1] Stavisky J. (2014). Too many cats: How owner beliefs contribute to overpopulation. Vet. Rec..

[2] Slater M.R. (2001). The role of veterinary epidemiology in the study of free-roaming dogs and cats. Prev. Vet. Med..

[3] Askew N.P., Vial F., Smith G.C. (2018). Status of urban feral cats Felis catus in England: A comparative study. bioRxiv.

[4] Sparkes A.H., Bessant C., Cope K., Ellis S.L.H., Finka L., Halls V., Hiestand K., Horsford K., Laurence C., MacFarlaine I. (2013). ISFM Guidelines on Population Management and Welfare of Unowned Domestic Cats (Felis catus). J. Feline Med. Surg..

[5] Gilhofer E.M., Windschnurer I., Troxler J., Heizmann V. (2019). Welfare of feral cats and potential influencing factors. J. Vet. Behav..

[6] Kreisler R.E., Cornell H.N., Levy J.K. (2019). Decrease in Population and Increase in Welfare of Community Cats in a Twenty-Three Year Trap-Neuter-Return Program in Key Largo, FL: The ORCAT Program. Front. Vet. Sci..

[7] Tan K., Rand J., Morton J. (2017). Trap-Neuter-Return Activities in Urban Stray Cat Colonies in Australia. Animals.

[8] Kilgour R.J., Magle S.B., Slater M., Christian A., Weiss E., DiTullio M. (2017). Estimating free-roaming cat populations and the effects of one year Trap-Neuter-Return management effort in a highly urban area. Urban Ecosyst..

[9] Longcore T., Rich C., Sullivan L.M. (2009). Critical Assessment of Claims Regarding Management of Feral Cats by Trap–Neuter–Return. Conserv. Biol..

[10] Miller P.S., Boone J.D., Briggs J.R., Lawler D.F., Levy J.K., Nutter F.B., Slater M., Zawistowski S. (2014). Simulating free-roaming cat population management options in open demographic environments. PLoS ONE.

[11] Mcdonald J.L., Farnworth M.J., Clements J. (2018). Integrating Trap-Neuter-Return Campaigns into a Social Framework: Developing Long-Term Positive Behavior Change Toward Unowned Cats in Urban Areas. Front. Vet. Sci..

[12] Stavisky J., Brennan M.L., Downes M., Dean R. (2012). Demographics and economic burden of un-ownedcats and dogs in the UK: Results of a 2010 census. BMC Vet. Res..

[13] Clark C.C.A., Gruffydd-Jones T., Murray J.K. (2012). Number of cats and dogs in UK welfare organisations. Vet. Rec..

[14] Aguilar G.D., Farnworth M.J. (2013). Distribution characteristics of unmanaged cat colonies over a 20 years period in Auckland, New Zealand. Appl. Geogr..

[15] Aguilar G.D., Farnworth M.J. (2012). Stray cats in Auckland, New Zealand: Discovering geographic information for exploratory spatial analysis. Appl. Geogr..

[16] Finkler H., Terkel J. (2012). The contribution of cat owners’ attitudes and behaviours to the free-roaming cat overpopulation in Tel Aviv, Israel. Prev. Vet. Med..

[17] Finkler H., Hatna E., Terkel J. (2011). The impact of anthropogenic factors on the behavior, reproduction, management and welfare of urban, free-roaming cat populations. Anthrozoos.

[18] Hobbs S.J., White P.C.L. (2012). Motivations and barriers in relation to community participation in biodiversity recording. J. Nat. Conserv..

[19] Pinillos R.G., Appleby M.C., Manteca X., Scott-Park F., Smith C., Velarde A. (2016). One Welfare—A platform for improving human and animal welfare. Vet. Rec..

[20] McLeod L.J., Driver A.B., Bengsen A.J., Hine D.W. (2017). Refining Online Communication Strategies for Domestic Cat Management. Anthrozoos.

[21] Attree P., French B., Milton B., Povall S., Whitehead M., Popay J. (2011). The experience of community engagement for individuals: A rapid review of evidence. Heal. Soc. Care Community.

[22] Mattis J.S., Hammond W.P., Grayman N., Bonacci M., Brennan W., Cowie S.A., Ladyzhenskaya L., So S. (2009). The social production of altruism: Motivations for caring action in a low-income urban community. Am. J. Community Psychol..

[23] Harkins C., Shaw R., Gillies M., Sloan H., Macintyre K., Scoular A., Morrison C., Mackay F., Cunningham H., Docherty P. (2010). Overcoming barriers to engaging socio-economically disadvantaged populations in CHD primary prevention: A qualitative study. BMC Public Health.

[24] Canvin K., Marttila A., Burstrom B., Whitehead M. (2009). Tales of the unexpected? Hidden resilience in poor households in Britain. Soc. Sci. Med..

[25] Ministry of Housing Communities & Local Government English Indices of Deprivation 2015. https://www.gov.uk/government/statistics/english-indices-of-deprivation-2015.

[26] Michie S., van Stralen M.M., West R. (2011). The behaviour change wheel: A new method for characterising and designing behaviour change interventions. Implement. Sci..

[27] NVivo Q.S.R. (2000). Qualitative Data Analysis Program.

[28] R Core Team R. (2014). A Language and Environment for Statistical Computing.

[29] Swarbrick H., Rand J. (2018). Application of a Protocol Based on Trap-Neuter-Return (TNR) to Manage Unowned Urban Cats on an Australian University Campus Helen. Animals.

[30] Schmidt P.M., Swannack T.M., Lopez R.R., Slater M.R. (2009). Evaluation of euthanasia and Trap-Neuter-Return (TNR) programs in managing free-roaming cat populations. Wildl. Res..

[31] Ross N. (2002). Community belonging and health. Heal. Rep..

[32] Hystad P., Carpiano R.M. (2012). Sense of community-belonging and health-behaviour change in Canada. J. Epidemiol. Community Health.

[33] Loss J., Lindacher V., Curbach J. (2014). Online social networking sites—A novel setting for health promotion?. Heal. Place.

[34] Bandura A. (1977). Self-efficacy: Toward a Unifying Theory of Behavioral Change. Psychol. Inq..

[35] Zhao H., Seibert S.E., Hills G.E. (2005). The Mediating Role of Self-Efficacy in the Development of Entrepreneurial Intentions. J. Appl. Psychol..

[36] Ajzen I. (1991). The theory of planned behavior. Organ. Behav. Hum. Decis. Process..

[37] Piliavin J.A., Siegl E. (2007). Health Benefits of Volunteering in the Wisconsin Longitudinal Study. J. Health Soc. Behav..

[38] Seligman M.E.P., Csikszentmihalyi M. (2000). Positive psychology: An introduction. Am. Psychol..

[39] Szolnoki G., Hoffmann D. (2013). Online, face-to-face and telephone surveys—Comparing different sampling methods in wine consumer research. Wine Econ. Policy.

